# Supplementary material for: Harms of introduced large herbivores outweigh benefits to native biodiversity
Source: Nat Commun. 2025 Sep 16;16:8260. doi: 10.1038/s41467-025-63807-2 (PMC12441147; doi:10.1038/s41467-025-63807-2)
Supplement: Supplementary file 1 — Supplementary Information [file 41467_2025_63807_MOESM1_ESM.pdf]

Supplementary information

Supplementary Table 1. Full sets of mechanisms through which alien species can cause negative and positive impacts on native species as outlined in EICAT[1] and EICAT+[2] and number of impact observations (N = 2021) assessed in this paper for each mechanism. Alien large mammalian herbivore (LMH) caused negative impacts to native species through 8 mechanisms and positive impacts through 7 mechanisms. For 1,896 impact observations, it was possible to unanimously assign impacts to a single mechanism, while for 125 observations more than one mechanism was assigned to the observed impacts. Numbers with superscript letters refer to cases where multiple mechanisms were simultaneously linked to a single observed impact. Direct mechanisms are underlined, while colours denote mechanisms through which both negative and positive impacts can be caused. Note that while chemical, physical, or structural impact on ecosystems were kept distinct in the original formulation of EICAT(+), here we have combined them following Volery et al. (2021)[3]. This decision reflects the reality that in most cases, a neat distinction between them was not elucidated in the impact report.

Mechanism	N. of impact observations	Total
EICAT		
1. Competition[a]	15, 2[g], 2[c]	19
2. Predation[b]	52, 1[d]	53
3. Hybridization	24	24
4. Transmission of disease[c]	4, 2[a]	6
5. Parasitism	0	0
6. Poisoning/toxicity	0	0
7. Bio-fouling or other direct physical disturbance[d]	196, 91[e], 8[e,g], 1[b]	296
8. Grazing/herbivory/browsing[e]	865, 91[d], 8[d,g], 18[f]	982
9. Chemical/Physical/Structural impact on ecosystem[f]	296, 18[e]	314
10. Indirect impact through interactions with other taxa[g]	45, 8[e,d],2[a]	55
EICAT+		
1. Provision of trophic resources	9	9
2. Overcompensation	12	12
3. Hybridization	8	8
4. Disease reduction	0	0
5. Dispersal facilitation	30	30
6. Epibiosis or other direct provision of habitat[h]	3, 6[j,i]	9
7. Chemical/Physical/Structural impact on ecosystem[i]	68, 6[h,j]	74
8. Indirect impact through interactions with other taxa[j]	269, 6[h,i]	275

Supplementary Table 2. Complete list of post hoc pairwise comparisons conducted to identify differences between levels of categorical predictors on the probability of an alien LMH species causing a strong impact on native biodiversity. Comparisons were conducted using Tukey's Honest Significant Difference correction for multiple comparisons, with two-sided tests at a 95% confidence level. S. cons.: secondary consumers; P. cons.: primary consumers; Produc.: producers; Decomp.: decomposers. Asterisks indicate significant differences: ***p<0.001; **p<0.01; *p<0.05.

**Predictor**	**Comparison**	**odds.ratio**	**SE**	**z.ratio**	**p.value**
Direction	Negative / Positive	2.11	0.587	2.693	0.0071**
Location	Island / Mainland	0.113	0.0648	3.806	0.0001***
Mechanism type	Direct / Indirect	0.343	0.126	-2.920	0.0035**
Trophic level	S. cons. / P. cons.	3.170	1.298	2.817	0.0250 *
	S. cons. / Produc.	1638	0.683	1.183	0.6375
	S. cons. / Decomp.	5.245	4.073	2.134	0.1423
	P. cons. / Produc.	0.517	0.237	-1.441	0.4736
	P. cons. / Decomp.	1.655	1.343	0.620	0.9256
	Produc. / Decomp.	3.203	2.571	1.450	0.4679
Direction * Location	Direction = Negative<br>Island / Mainland	0.0814	0.0459	4.452	<0.0001***
	Direction = Positive<br>Island / Mainland	0.1574	0.1033	2.818	0.0048**
Confidence	Low / Medium	0.1977	0.0597	-5.370	<0.0001***
	Low / High	0.0644	0.0256	-6.889	<0.0001***
	Medium / High	0.3257	0.1130	-3.232	0.0035**
Direction * Confidence	Direction = Negative<br>Low / Medium	0.8168	0.2370	-0.698	0.7648
	Direction = Negative<br>Low / High	0.1578	0.0771	-3.779	0.0005***
	Direction = Negative<br>Medium / High	0.1932	0.0883	-3.597	0.0009***

	Direction = Positive<br><br>Low / Medium	0.0463	0.0235	-6.196	<0.0001***
	Direction = Positive<br><br>Low / High	0.0253	0.0138	-6.908	<0.0001***
	Direction = Positive<br><br>Medium / High	0.5472	0.2929	-1.124	0.4993

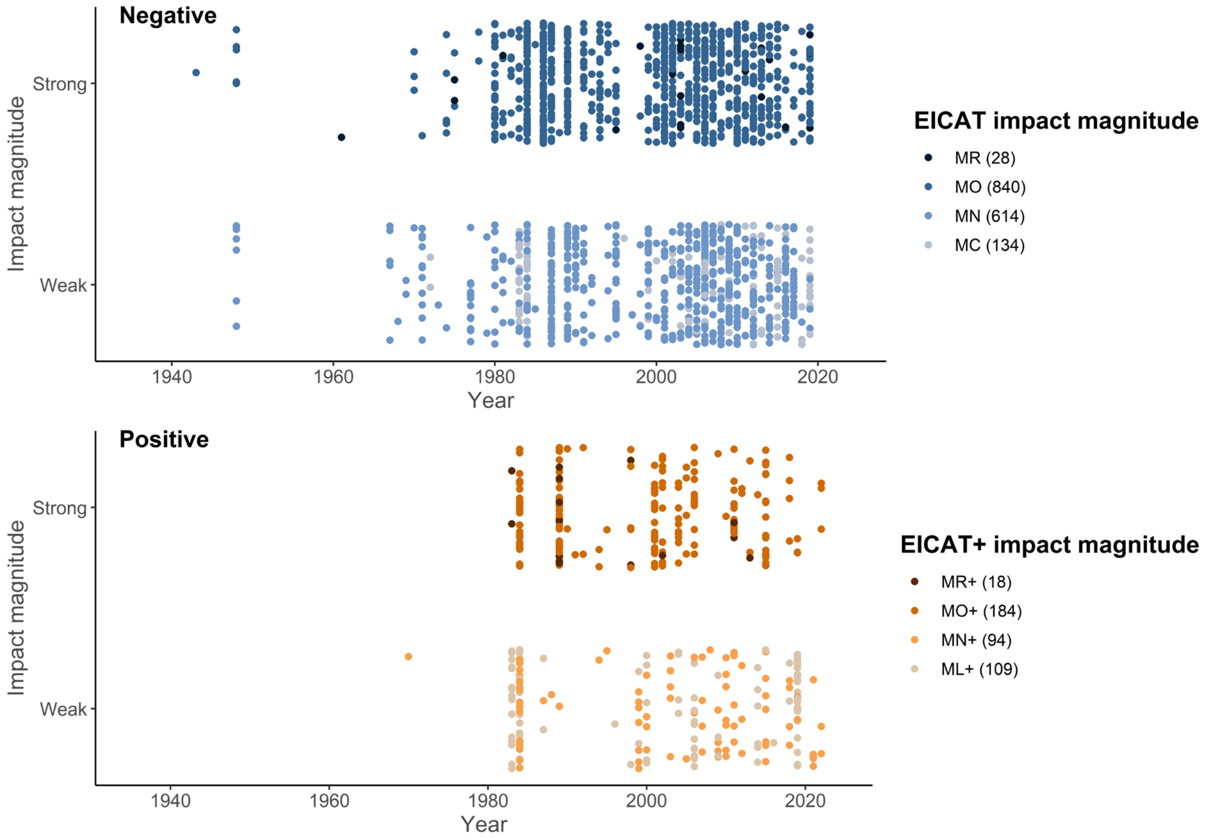

Supplementary Fig. 1: Temporal distribution of negative and positive impacts as classified under EICAT(+) across introduced LMH species

Dots represent single observations of negative (blue) and positive (orange) impacts having different impact magnitude (strong vs. weak) across years, with a jitter function to prevent overlap. Numbers in brackets represent the sample size for each group. Note that no impact observation, whether positive and negative, was assigned a "Massive" level of magnitude.

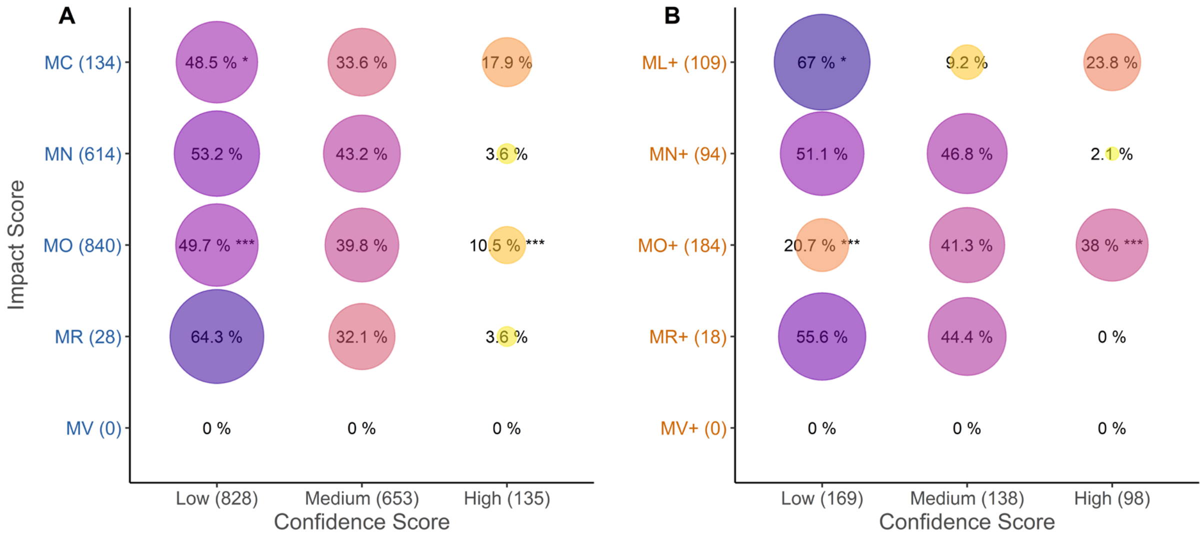

Supplementary Fig. 2: Confidence assigned to different impact scores under EICAT(+) across introduced LMH species

Percentage of impact observations assigned with different confidence with EICAT (A) and EICAT+ (B) at each level of impact magnitude. Asterisks indicate cases where the percentages differed significantly between negative (A) and positive impacts (B), as determined by pairwise Z-tests. P-values: ***p<0.001; *p<0.05. Numbers in brackets represent the sample size for each group.

Supplementary References

1. IUCN. IUCN EICAT Categories and Criteria. The Environmental Impact Classification for Alien Taxa. IUCN EICAT Categories and Criteria: First Edition. (2020). doi:10.2305/IUCN.CH.2020.05.en.

2. Vimercati, G. et al. The EICAT+ framework enables classification of positive impacts of alien taxa on native biodiversity. *PLOS Biology* 20, e3001729 (2022).

3. Volery, L., Jatavallabhula, D., Scillitani, L., Bertolino, S. & Bacher, S. Ranking alien species based on their risks of causing environmental impacts: A global assessment of alien ungulates. *Global Change Biology* 27, 1003–1016 (2021).
