# Supplementary material for: Harms of introduced large herbivores outweigh benefits to native biodiversity
Source: Nat Commun. 2025 Sep 16;16:8260. doi: 10.1038/s41467-025-63807-2 (PMC12441147; doi:10.1038/s41467-025-63807-2)
Supplement: Supplementary file 3 — Description of Additional Supplementary Information [file 41467_2025_63807_MOESM3_ESM.pdf]

Description of Additional Supplementary Files

File Name: Supplementary Data 1
Description:
- General Dataset – Contains the complete dataset used in the manuscript.

- EICAT+ assessed data – Lists all recorded positive environmental impacts, along with additional information, assessed with the EICAT+ framework.

- EICAT assessed data – Lists all recorded negative environmental impacts, along with additional information, assessed with the EICAT framework.
