# Supplementary material for: Harms of introduced large herbivores outweigh benefits to native biodiversity
Source: Nat Commun. 2025 Sep 16;16:8260. doi: 10.1038/s41467-025-63807-2 (PMC12441147; doi:10.1038/s41467-025-63807-2)
Supplement: Supplementary file 5 — Reporting Summary [file 41467_2025_63807_MOESM5_ESM.pdf]

nature portfolio

Corresponding author(s): NCOMMS-24-53027

Last updated by author(s): Jul 9, 2025

Reporting Summary

Nature Portfolio wishes to improve the reproducibility of the work that we publish. This form provides structure for consistency and transparency in reporting. For further information on Nature Portfolio policies, see our Editorial Policies and the Editorial Policy Checklist.

Statistics

For all statistical analyses, confirm that the following items are present in the figure legend, table legend, main text, or Methods section.

n/a	Confirmed	
☐	☒	The exact sample size (n) for each experimental group/condition, given as a discrete number and unit of measurement
☒	☐	A statement on whether measurements were taken from distinct samples or whether the same sample was measured repeatedly
☐	☒	The statistical test(s) used AND whether they are one- or two-sided<br>*Only common tests should be described solely by name; describe more complex techniques in the Methods section.*
☐	☒	A description of all covariates tested
☐	☒	A description of any assumptions or corrections, such as tests of normality and adjustment for multiple comparisons
☐	☒	A full description of the statistical parameters including central tendency (e.g. means) or other basic estimates (e.g. regression coefficient) AND variation (e.g. standard deviation) or associated estimates of uncertainty (e.g. confidence intervals)
☐	☒	For null hypothesis testing, the test statistic (e.g. F, t, r) with confidence intervals, effect sizes, degrees of freedom and P value noted<br>*Give P values as exact values whenever suitable.*
☒	☐	For Bayesian analysis, information on the choice of priors and Markov chain Monte Carlo settings
☐	☒	For hierarchical and complex designs, identification of the appropriate level for tests and full reporting of outcomes
☒	☐	Estimates of effect sizes (e.g. Cohen's d, Pearson's r), indicating how they were calculated

Our web collection on statistics for biologists contains articles on many of the points above.

Software and code

Policy information about availability of computer code

Data collection	Data were manually collected by using search strings in Google Scholar to conduct a literature review. No specific software was used to extract data.
Data analysis	Data was analyzed with the statistical program R using its interface "R Studio". The versions of the R packages used are specified both here and in the text: package “BSDMA” (version 1.2.2); package “emmeans”(version 1.8.6); package “lme4” (version 1.1.35.1); R package “DHARMa” (version0.4.6.54); package “ggplot2” (version 3.4.2); package “sjPlot” (version 2.8.15).<br>The R scripts used in this study for statistical analyses and data visualization, along with the versions of the R packages, are publicly available in a Figshare repository at the following link: https://doi.org/10.6084/m9.figshare.28046465.v1

For manuscripts utilizing custom algorithms or software that are central to the research but not yet described in published literature, software must be made available to editors and reviewers. We strongly encourage code deposition in a community repository (e.g. GitHub). See the Nature Portfolio guidelines for submitting code & software for further information.

Data

Policy information about availability of data

All manuscripts must include a data availability statement. This statement should provide the following information, where applicable:

- Accession codes, unique identifiers, or web links for publicly available datasets
- A description of any restrictions on data availability
- For clinical datasets or third party data, please ensure that the statement adheres to our policy

The complete dataset assembled and used in this study is provided in the Supplementary Data 1. All intermediate datasets used to conduct the analyses and to generate the figures and results are publicly available via Figshare at https://doi.org/10.6084/m9.figshare.28046465.v1.

Research involving human participants, their data, or biological material

Policy information about studies with human participants or human data. See also policy information about sex, gender (identity/presentation), and sexual orientation and race, ethnicity and racism.

Reporting on sex and gender	n/a
Reporting on race, ethnicity, or other socially relevant groupings	n/a
Population characteristics	n/a
Recruitment	n/a
Ethics oversight	n/a

Note that full information on the approval of the study protocol must also be provided in the manuscript.

Field-specific reporting

Please select the one below that is the best fit for your research. If you are not sure, read the appropriate sections before making your selection.

☐ Life sciences ☐ Behavioural & social sciences ☒ Ecological, evolutionary & environmental sciences

For a reference copy of the document with all sections, see nature.com/documents/nr-reporting-summary-flat.pdf

Ecological, evolutionary & environmental sciences study design

All studies must disclose on these points even when the disclosure is negative.

Study description

We used the IUCN EICAT framework and the EICAT + framework to systematically assess negative and positive impacts of introduced large mammalian herbivores to native biodiversity on a global scale and to investigate to what extent insularity and trophic position shape the magnitude of both negative and positive impacts experienced by native species.

1) Impact observations were collected by following established search protocols. Each impact observation recorded refers to a specific alien large mammalian herbivore species at a specific location and year,along with one impacted native species, the assigned impact magnitude through EICAT/EICAT+, mechanism (direct or indirect), and associated confidence level. Additional information, such as the reference of the impact observation, year of impact (publication year when no specific year was given in the report), taxonomy and trophic level of the impacted native species (decomposer, producer, primary consumer, and secondary consumer/omnivore), geographical details (including precise coordinates,country's sub-unit such as district, state, region or county, country, continent, mainland or island) was collected.
2) One-sided paired sign tests were conducted to investigate whether the number of negative impacts consistently differed from that of positive impacts in species and reports exhibiting bidirectional impact observations.
3) Pairwise z-tests were conducted to investigate whether the proportion of impact observations assigned with low, medium, and high confidence differs between negative and positive impacts at each level of impact magnitude.
3) Generalized linear mixed-effect models (GLMMs) with binomial error distribution were built to test the effects of multiple predictors on impact magnitude, or more precisely the probability of an alien species causing a strong impact on native biodiversity. The response variable was the impact magnitude coded as “0” for “weak” and “1” for “strong” impacts, for both positive and negative impacts. The predictors coded as fixed effects were impact direction (positive vs. negative), the trophic level of the impacted species (four categories), the impact location (island vs. mainland), reporting impact year(scaled to 0 mean and 1SD), mechanism types (direct vs. indirect), and all 2-way interactions of the previous variables with impact direction. The Report ID and alien species name were included as random effects to account for pseudoreplication resulting from multiple observations from the same report and/or the same alien species. Additional GLMMs were computed on the same response variable by using the same predictors with the addition of a binomial predictor designating the introduction location as either "true alien" or "potential reintroduction to former native area" in accordance with the "refugee species concept".
4) Generalized linear mixed-effect models (GLMMs) with binomial error distribution were additionally constructed to test whether

weak and strong impacts were assigned with different levels of confidence,and to examine how the assignment of confidence varies by direction and over the years.

Research sample	Among the currently recognized 286 modern species of LMH (Cetartiodactyla,Perissodactyla, Proboscidea), including wild and domesticated forms 43 , we focused on 66 species from six families that have established alien populations according to the IUCN Global Register of Introduced and InvasiveSpecies (http://www.griis.org). We found 304 reports describing 1616 negative and 405 positive impacts for native species that could be classified under EICAT or EICAT+, from 29 of the 66 listed alien species. Negative and positive impacts were caused by 28 and 21 LMH species, respectively. About two thirds of alien species (20 out of 29) caused simultaneously both negative and positive impacts, although for species having bidirectional impacts, we detected 3.7 times more negative than positive impact observations overall (1489 vs. 399)
Sampling strategy	By following the data collection strategy explained in the section below, we have aimed to sample the highest number of observed impacts (both positive and negative) that introduced large mammalian herbivores have had on native biodiversity worldwide. Such impact data have been assessed through EICAT and EICAT+. Assessed impact data have then been used in the statistical analysis.
Data collection	We followed the search protocol described by Evans et al. to collect the data and built upon the work of Volery et al. by incorporating positive impacts of alien large mammalian herbivores on native species. The data sources were obtained by conducting a search using the following terms ('invasive' OR 'invasive species' OR'introduced species' OR 'introduced' OR 'alien' OR 'non-native' OR 'non-indigenous' OR 'feral' OR 'exotic' OR'positive impact' OR ' beneficial' OR 'benefit' OR 'positive effect' AND '[scientific name of the alien species]') in the online database Google Scholar (https://scholar.google.com) including articles published in scientific journals as well as grey literature, such as conference abstracts, governmental papers, and private sector research. Similar to Volery et al. a literature review was performed for all 66 alien large mammalian herbivore species. Data sources containing observed impacts of an alien species on a native population were selected based on the evaluation of the title, abstract, and content of the first 100 records found. Additionally, we followed up all references to other data sources with observed impacts in the selected papers until no additional impact records were found. The references gathered for negative impacts by Volery and coworkers were cross-checked to identify any additional positive impacts. Only observed impacts were included for classification, while potential or inferred impacts were not considered, in line with the guidelines of the frameworks used. Data was collected by Zoé Bescond--Michel and reviewed by Giovanni Vimercati and Sven Bacher.<br><br>Evans T, Kumschick S, Blackburn TM (2016) Application of the Environmental Impact Classificationfor Alien Taxa (EICAT) to a global assessment of alien bird impacts. Divers Distrib 22:919–931<br><br>Volery L, Jatavallabhula D, Scillitani L, Bertolino S, Bacher S (2021) Ranking alien species based ontheir risks of causing environmental impacts: A global assessment of alien ungulates. Glob ChangeBiol 27:1003–1016
Timing and spatial scale	Impact data is not restricted by time or space. It therefore includes all available historical records until 2022-2023 and is collected at the global scale. Specific year of report and region of impact for each impact observation can be found in the source data file provided with this paper.
Data exclusions	Only observed impacts found in the literature were used for assessment and statistical analysis, while potential or inferred impacts were not considered, in line with the guidelines of the frameworks used.
Reproducibility	To ensure reproducibility, all R codes and intermeditate datasets used to conduct the analyses and to generate the figures and results are publicly available via Figshare at https://doi.org/10.6084/m9.figshare.28046465.v1.
Randomization	The Report ID and alien species name were included as random effects to account for pseudoreplication resulting from multiple observations from the same report and/or the same alien species.
Blinding	Our study does not include any sensitive information requiring blinding.

Did the study involve field work? ☐ Yes ☒ No

Reporting for specific materials, systems and methods

We require information from authors about some types of materials, experimental systems and methods used in many studies. Here, indicate whether each material, system or method listed is relevant to your study. If you are not sure if a list item applies to your research, read the appropriate section before selecting a response.

Materials & experimental systems

n/a	Involved in the study	
☒	☐	Antibodies
☒	☐	Eukaryotic cell lines
☒	☐	Palaeontology and archaeology
☒	☐	Animals and other organisms
☒	☐	Clinical data
☒	☐	Dual use research of concern
☒	☐	Plants

Methods

n/a	Involved in the study	
☒	☐	ChIP-seq
☒	☐	Flow cytometry
☒	☐	MRI-based neuroimaging

Plants

Seed stocks	N/A
Novel plant genotypes	N/A
Authentication	N/A
